# Examining the Coping Response to Peer Relational Aggression Victimization

**DOI:** 10.1155/2011/473980

**Published:** 2011-04-04

**Authors:** Melissa M. Gomes

**Affiliations:** Department of Family and Community Health Nursing, School of Nursing, Virginia Commonwealth University, P.O. Box 980567, Richmond, VA 23298-0567, USA

## Abstract

*Purpose*. Relational aggression, rumor spreading, backstabbing, and social isolation, is psychologically damaging for adolescent girls. The purpose of this study was to provide an explanation of victimization response after experiencing peer relational aggression victimization. *Methods*. Grounded theory techniques were used to gain an understanding of the victimization experience and the coping responses used. *Findings. A theory of coping after experiencing peer relational aggression victimization* was generated. Girls voiced feelings of hurt and anger after the experience and expressed the following ways of coping as a result: distancing from others, retaliation against the aggressor, discussing their feelings with friends and family, writing their feelings down, and/or confronting the aggressor. *Clinical Implications*. Nurses should be aware of the phenomenon and asses, for incidences of relational aggression victimization so that they may provide strategies to assist the adolescent and her family with positive coping mechanisms in order to prevent maladaptive responses.

## 1. Introduction

Relational aggression, rumor spreading, backstabbing, and social isolation, is recognized as a violent act and is no longer considered a normal part of growing up. Research has indicated the experience of peer (girl-girl) relational aggression—whether from the standpoint of the perpetrator or the victim—as detrimental, resulting in serious consequences for those involved [1–4], indicating that it reverberates in a manner similar to physical aggression. Although relational aggression victimization is experienced by both boys and girls [5], it is a psychosocial experience that damages what matters most to girls, which is a connection to the peer group [6]. As an aspect of the bullying phenomena, it seeks to damage peer relationships through rejection, isolation, and character assassination. However, relational aggression is covert and often goes unnoticed to those not immersed within the social network. For girls, this is crucially important because it impacts social connections that are a vital aspect of growth and development, and self-identity formation [7]. Moreover, the maladaptive responses of depression, social anxiety, social phobia, borderline personality disorder, psychosocial maladjustment and decreased life satisfaction have all been associated with the relational aggression victimization experience [2, 8, 9]. Furthermore, an increase from previous years in the rates of suicide attempts in seemingly healthy African American female adolescents (Joe, Baser, Neighbors, Caldwell & Jackson, 2009) suggests a need to explore for varying predeterminants to suicidality, and PRAV may be a contributing factor. In light of this, we need more information about what happens following the victimization experience from those who are victimized. The purpose of this study was to provide an explanation of the response pattern using grounded theory techniques in order to gain a deeper understanding of the peer relational aggression victimization experience and its consequences.

## 2. Methods

### 2.1. Participants and Procedure

This qualitative report was part of a larger study that used a mixed method approach to explore the peer relational aggression victimization experiences of African American girls at a historically black college in the Southeastern United States. The Institutional Review Board at the sponsoring university approved the study prior to commencement of study activities. A detailed description of the quantitative report has been previously published [10]. In line with the overall purpose of the study, an African American female sample was chosen due to the paucity of information in the literature related to the experience in this population. The within-methods triangulation design was accomplished using a sequential implementation technique in which the participants were required to complete the quantitative aspect of the study prior to the qualitative portion. During the quantitative portion of the study, we explored the relationship between peer relational aggression victimization, depression, and self-esteem [10]. For the qualitative portion of the study, grounded theory techniques [11] were used to explore the response to the victimization experience through focus group interviews with a random selection of girls from the quantitative portion of the study (*n* = 15). Inclusion criteria for focus group participation was that the participant had to be an adolescent female no more than one year removed from attending high school, self-identified as Black or African American, and have a peer relational aggression victimization subscore on the Self Report of Aggression and Social Behavior Measure (SRASBM) [9] used during the quantitative phase of the study. The mean age of the participants in this study was 18.3. The purpose of this paper is to report solely on the qualitative outcomes of the analysis.

Following collection of the quantitative data, a random selection of twenty percent (*n* = 60) of the overall sample (*N* = 241) was invited to participate in a focus group session. Random selection was achieved by placing the corresponding participant numbers on slips of paper, which were then randomly drawn from the container, and the corresponding participant was invited to participate via the telephone. Of those who were randomly selected, 24 were able to be contacted and agreed to participate and 15 attended one of four focus group sessions. Focus groups were conducted to explore the girls' response after experiencing peer relational aggression victimization. To provide a convenience for the girls, focus group sessions were held—in an education building on the main campus and in the dormitory. Small focus group sessions with groups of 3-4 girls were utilized to provide a comfortable and trusting environment in which there was a greater opportunity to share personal feelings and experiences [12]. Focus groups were held until data saturation was achieved. Four, two-hour focus group sessions were conducted with a total of 15 girls. The principal investigator was trained and possessed the ability to monitor the group process in order to be aware of key interactions among the group that could have impact on the data obtained. Written informed consent was obtained from all the girls before the focus group sessions began. The principal investigator conducted the focus groups along with three trained senior undergraduate research assistants whose primary responsibility was to take notes related to the discussions during the focus group sessions. In the event any of the girls needed assistance as a result of the focus group discussions (none did), a nurse psychotherapist and a doctorally prepared counselor were on hand and able to be contacted by telephone immediately. All sessions were audio taped for transcription and analysis.

During the focus group sessions, semistructured interviewing was done using an interview guide to facilitate the process. The interview guide was designed to provide a direction for the focus group sessions by beginning with a vignette (see vignette; Section 6.1) designed to stimulate the memory of the participants pertaining to their own similar experience with peer relational aggression victimization. The vignette was used as they have demonstrated effectiveness in obtaining information about a person's experience with similar situations and scenarios. To assist the participants with opening up about their experiences, the interview guide contained the following types of questions: opening, introductory, transition, key, and ending. The interview guide proved essential in the process, in that it provided the direction of the focus group session by providing a set of questions and topics to be covered in the session. Along with the main questions, probing responses were used when warranted to encourage the participants to open up about their experiences [13].

### 2.2. Data Analysis

Qualitative analysis of the focus group findings was conducted using grounded theory techniques [11]. Open coding, axial coding, and selected coding were done in tandem to provide an explanation of the response to the victimization experience. Transcripts from each session were analyzed to explore for similar concepts, which indicated a pattern of response, while a connection between the categories was explored through the comparison of categories of data across the focus group sessions to establish a theory of response after peer relational aggression victimization experience. In addition, two other experienced, African American, qualitative researchers independently identified themes and validated the resultant themes that were identified by the principal investigator.

### 2.3. Findings

**Figure 1:**
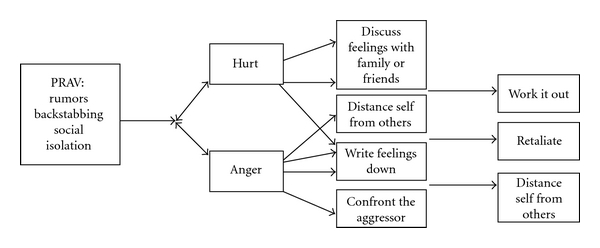
Theory of coping after experiencing peer relational aggression victimization.

*A theory of coping after experiencing peer relational aggression victimization* was generated, see Figure 1. There were nine overall themes that emerged from the focus group sessions, two indicating their initial response of hurt and anger and seven themes indicating their coping responses. The following are some excerpts from selected girls in the study, which help to illuminate the themes. All names have been changed to protect confidentiality of the girls.

### 2.4. Reactive Responses

Upon reflecting on her experiences, Shannell reported feeling a sense of hurt as a result of not being included among her friends. She reported “There were a couple of girls, I felt, I could tell them anything. I told them some stuff that meant a lot to me—like what was going on with my family and stuff, and why we moved … just all kind of stuff, and this stuff was deep to me. I thought they would, you know, keep it confidential, and they did not. They just told people and then other people started treating me differently and stuff.” In this instance, Shannell discussed an occasion in which she learned she could not trust secrets related to why her family moved with the girls she thought were her friends. Her confidence in their friendship was betrayed and her ability to trust others was compromised. For her, the experience had a distressing and rippling effect, causing others to think about her and her family differently. 

#### 2.4.1. Hurt

Other girls in this study voiced feelings of hurt after the victimization experience, but it was most poignant, and deeply felt, when the aggressor was thought of as a close friend. Feelings of sadness were palpable as some of the girls voiced a shattered confidence in their close friends as a result of the victimization experience. Tammy stated “[It] hurt deep—'cause I felt I could put my trust in them. 'Cause I'm a trusting person quickly, and I seem to gain friendships quickly. And so when I trust you with something and I expect you to keep it between us if I say keep it between us.”

Although still pained, when the aggressor was not thought of as a close friend, the victimized girl expressed a readiness to move on by adopting new friendships—despite feeling hurt by the experience. Kenya, reflecting upon on her own experience said “I didn't want to show them like face-to-face how I felt—because I knew they had done it intentionally to hurt my feelings. So like I just adapted to my new friends and basically put it behind me, 'cause there was nothing more that I could do.” 

#### 2.4.2. Anger

Although not all the girls verbalized the response, Talia's comments suggested the outrage she felt once she found out other girls were talking about her behind her back to one of her friends. She recalled “I would get angry at my friend because she was just letting it happen. It made me feel bad … kind of angry because I never did that to her.”

### 2.5. Coping Response

The experience of peer relational aggression victimization was felt as a negative experience, one that required a response. All of the girls discussed coping methods even though their responses indicated a deep sense of violation and a desire to protect themselves from future incidences. 

#### 2.5.1. Distancing from Others

The coping response of distancing from others was voiced as a way for the girls to protect themselves from the hurt of repeat victimization. From the stories shared, it appeared that the girls developed a protective wall to guard against future hurt and it changed the way in which they interacted with others in developing future friendships.

After becoming a victim of a backstabbing incident Tammy expressed her tentativeness for making friends. She said “From my past experience, it is kind of hard to call everyone my friend now … I do not take the word friend lightly … once you hurt me one time … I am going to feed you with a long spoon … that is saying I am going to keep my distance.”

 She also reiterated her self-protection by saying “It's really hard for me to make friends like that. Like it takes me some time and I don't know whether it's me—I can't do that.” 

The girls in the group verbalized learning from observing interactions among other girls, and the idea of trusting a group of girl peers was mentioned as not an easy thing to do. Shanese noted “I am just careful what I say around people … there's just a lot of people who I don't say stuff to because I see how others do people … talk about your other friends behind your back … it is really sad; you have to watch what you say.”

As a result of previous negative female peer interactions Shanese verbalized the realization “I can't trust everybody and everybody is not gonna be my friend. Now, it takes some time for me to trust—[people] have to grow on me, I need to figure out who they are first.”

Past experiences with peer relational aggression victimization caused some of the girls in the group to hold back on their interactions with others. Shelly said “I'm concerned about what somebody might think of me and how I feel … and so sometimes I hold back.”

#### 2.5.2. Retaliation

The coping response of retaliation was voiced as a way of empowering herself after the victimization experience. It appeared that some of the girls used this coping response to make a previous aggressor feel the hurt she felt during the initial victimization experience. This strategy was discussed as a protective mechanism, one that would prevent the adolescent girl from appearing weak and becoming the host of repeat victimization experiences. Danielle noted, “I turned the tables on her and did to her what she did to me … I didn't feel good 'cause I saw her face when she walked away but …  well, I guess she knows how I feel now.”

Anessa, reflecting on the use of rumor spreading, backstabbing, and social isolation among her peer group, admitted “Oh, yeah. Yeah. I've been a victim of it and I've been a giver of it. I'm on the giving end and the receiving end.”

#### 2.5.3. Expressing Feelings with Friends, Family, or Self

Seeking social support after relational aggression victimization is a coping strategy that may help to preserve the friendship by allowing the victim to vent feelings to others, without having to endure an open conflict with the aggressor. The expression of feelings was articulated as a way to get out the emotions felt after the experience. Some of the girls expressed being able to talk with other friends about what they were going through. Others felt like it was better to discuss their feelings with family members.

Shanese recounted learning how to positively deal with the experience. She stated “As I grew older, I've learned to talk about it. It's really good to talk about things because holding it inside is not gonna solve any problems. Even if you don't talk about it to the person or confront the person, tell someone you know.”

After thinking about her experience with peer relational aggression victimization, Tanesha stated “[I told] my mom. I tell my mom everything. Like we're really close. We're like sisters … It helped a lot because she'll usually tell me ‘you know who you are, we know who you are.'”

#### 2.5.4. Coping by Confronting the Aggressor

Coping by confronting the aggressor was a way in which the adolescent girl felt she could deal with the problem in a forthright manner. Not all of the girls were able to voice this coping response. However, those that voiced this response also projected an air of self-confidence and assurance despite the victimization experience. The ability to use this coping response seemed to coincide with the level of closeness between the aggressor and the victim. Tammy said “I would approach them. I would try to be as calm as possible, even though I may be mad about it.” Shanell mentioned her reasons for confronting an aggressor. She stated “If I don't confront them, it's gonna bother me. It's gonna keep nagging at me and I'm gonna wonder, you know, what's going on? Why was she talking about me?

#### 2.5.5. Working It Out

The coping response of trying to work it out with the aggressor seemed to depend upon the level of closeness between the aggressor and the victim. Some of the girls voiced the willingness to work it out with the aggressor if they felt they had a history with the individual that needed to be preserved. In this study, feelings of trying to work it out coincided with wanting to maintain a sense of friendship between the aggressor and the victim. When discussing her reasons for wanting to work it out, Jaelle noted “Me being the person I am, I'm gonna show her what a real friend is … I'm gonna show her 'cause she does not know how to be a real friend like me. So I try and help my friends like build them up. I try and show them like, you know, that it doesn't have to always be this way. It doesn't have to be like that [relationally aggressive].”

## 3. Discussion

The literature suggests that peer relational aggression victimization is a substantial predictor of social anxiety and depression in adolescents [1, 14]. However, it is important to take note of the girls in this study that displayed factors of resilience, which described their ability to overcome the victimization experience through the incorporation of a variety of coping mechanisms—which is a key component of resilience theory. They were able to identify their own strengths, which allowed them to succeed despite encountering adversity through the exposure to a psychosocial stressor [15, 16]. As indicated by the themes presented in this paper, factors must exist internally (self-esteem, coping skills, spirituality) and externally (multilevel attachments with family, school, community, church) to help the impressionable adolescent girl overcome adversity. 

If not dealt with effectively, chronic interpersonal stress impacts coping ability placing an increased demand on coping abilities leading to a depletion of coping resources and resulting in maladaptive outcomes [17]. Researchers have categorized coping mechanisms according to their engagement with the risk, while engaging with a risk includes trying to work it out through emotion expression or rumination and disengaging includes removing oneself from the situation as with denial, and wishful thinking [18]. These paths were evident among the girls in this study. 

After the initial response of hurt and/or anger, a variety of coping mechanisms was utilized. In this study, the path chosen and final outcome were dependent upon the presentation of the incident, or the relationship between the aggressor and victim. It could be reasoned that some of the girls in the study foresaw this potential pitfall, inasmuch as they chose not to discuss their emotions openly with anyone, but rather chose to write them down. Previous research has indicated that the level of hurt the adolescent girl experiences as a result of being a victim of relational aggression is in direct relation to the level of closeness shared by the aggressor and victim [19], such that the responses of hurt and anger as a result of the relational aggression victimization experience are highly correlated, thereby making them difficult to differentiate when discussing the feelings that resulted from the experience. In their comments, it was evident that indications of altering their friendship styles were discussed as a consequence of the experience. This method of coping could perhaps further perpetuate the cycle of aggression by providing information about the situation that others could circulate as a rumor. It appeared that different scenarios called for different methods. Essentially, the amount of information divulged seemed to depend upon the amount of trust the individual had in the person she was confiding in at the time. Information-sharing choices also hinged on where the girl was in her emotional response to the victimization experience, which has also been reported in previous research among younger adolescent girls [20]. 

As indicated by the coping responses chosen in response to the victimization experience, coping patterns can be positive or negative based upon the individual's possession of coping skills. Because coping skills are a learned response and continue as a pattern into adulthood [18], it is crucial that adolescent girls faced with this victimization experience learn positive ways of coping to prevent the use of ineffective coping skills such as retaliation.

## 4. Limitations

This study had limitations. Although we knew that all the girls in the focus groups did have a victimization score as required in the inclusion criteria for participation, their level of victimization was uncertain. Therefore, it is possible that some of the girls that participated in the focus groups had more coping skills and were better able to verbalize their feelings as a result of the experience. In addition, the sample of girls used in the study were older adolescents who had embarked on a college experience and it is possible that they possessed different resources and skills than girls who were not able to attend college and thus had a more supportive infrastructure which allowed them to respond differently to the relational aggression experience. 

### 4.1. Clinical Implications

While many of us have been taught “sticks and stones will break our bones but words will never hurt”; it has been demonstrated that the negative experience of peer relational aggression is as equally damaging as physical aggression and can have long-lasting effects beyond the initial experience, affecting feelings of self and future relationship development [2]. This study lends credence to the overall argument that this experience alters the formation of female bonds via the social isolation experience initiated in the aggressive act, as well as by the self-imposed isolation response. The prolonged experience of these feelings could lead to future physical and emotional problems for the victim, as suggested in previous research. The prolonged or repeated experience of peer relational aggression victimization can also directly impacts a girl's of self-esteem, such that the more she is victimized, the lower her self-esteem will fall. Ironically, this may further predispose her to the victimization experience. While most of the girls in the this study were able to verbalize either their feelings or coping responses as a result of the peer relational aggression victimization experience, this verbalization was essential in identifying areas in need of assistance after the victimization experience and nurses should be aware that some girls may not be able to do so. Therefore, primary health care providers, school nurses, and college health services must be ready to asses for peer relational aggression victimization so that they may provide strategies to assist the girl and her family with identifying and fostering positive coping responses through the use of self-awareness via mindfulness-based attention activities [21, 22] which will help to decrease the ruminative effect of the victimization experience as well as create an opportunity to tap into personal strengths to help combat the negativity related to victimization experience. In addition, health care providers should continue to stress the importance of conflict resolution skills among adolescents and be available to identify positive methods for resolving social stressors which are amenable to the adolescent mores and beliefs while stressing the importance of overall health and well-being through the use of a variety of coping methods including physical exercise and mental awareness [23]. In doing so, these preventive strategies will serve to facilitate the prevention of maladaptive responses such as depression related to the internalization of negative feelings as a result of the experience.

### 4.2. Research Implications

Despite the gaining knowledge concerning relational aggression, more information is needed to ascertain why some girls who are victimized are able to bounce back unaffected and why some girls develop maladaptations. Research is needed to tap into the different responses so that the girls who are at risk can be taught strategies to build their support structure once faced with this experience. In addition, more longitudinal work is needed to build the science and establish this victimization experience as one that requires policy changes in the way we treat this phenomena.

## 5. Conclusion

This study lends credence to the overall argument that the experience of peer relational aggression victimization alters the formation of female bonds via the social isolation experience initiated in the aggressive act, as well as by the self-imposed isolation response. We must be mindful of the impact this experience can have on the individual and no longer dismiss it as benign. Although some girls may be able to respond positively and move past the victimization, those who cannot are at risk of poor health outcomes.

## 6. Focus Group Interview Guide

### 6.1. Vignette

Jasmine and Alisha have been best friends since the second grade. They have lived in the same neighborhood and been on the same community cheerleading squad together for as long as they can remember. They are now fourteen years old and are excited to be freshmen in high school. They have been looking forward to this moment in time for so long! Ever since they were in junior high school they talked about joining the high school junior varsity cheerleading squad together. This year is their year! 

Ultimately, they both tried out for the cheerleading squad together. However, only Alisha makes the squad, Jasmine does not. Because of this Jasmine and Alisha are now not on the same cheerleading squad for the first time ever! And consequently, a new girl who is on the cheerleading squad with Alisha has entered the picture. At first, all three of the girls Jasmine, Alisha, and Mia were friends and they hung out together. However lately, Mia tells Alisha that she does not want to be around Jasmine anymore because she is not cool enough for them. After all, she didn't make the cheerleading squad so why should they be associated with her. Mia believes they have more popular people to socialize with. As a result, Alisha and Mia have been doing more things together after cheerleading practice and leaving Jasmine out of their activities. It has gotten to the point where they do not invite Jasmine to hang with them anymore. In fact, when Jasmine sees them in school and tries to approach them, they turn the other way, huddle together and laugh while looking at Jasmine and sometimes they even act like she is not there. Jasmine feels very lonely and longs for the best friend she thought she had. Lately, Jasmine has been sitting alone at lunch and walking home by herself, while Alisha gets to sit at the lunch table with all of the other cheerleaders and athletes. This makes Jasmine very upset because she doesn't know why Alisha and Mia do not want to be her friend anymore. She cannot recall doing anything to make them mad.

**Description of Experience**

Tell us about a time when you experienced relational aggression in high school?

Probe: When did this happen?
Probe: What happened?
Probe: How did this make you feel?
Probe: What did you do after this happened?
Probe: How did you deal with your feelings as a result?
Probe: Why do think she did this to you?
Probe: Did you talk to anyone else about this?

How were you viewed by the other girls in your social network before this happened? After it happened?

Probe: Do you think your interaction with other females has been affected as a result of your experience?
Probe: Do you think that something you did brought on the aggression?

How did others in the social group view the girl who did this before it happened? After it happened?

Probe: Was this a way to gain popularity?
Probe: Was this a way to gain a stronger influence in the peer group?

Do these situations still occur in college life?
